# Letter to the Editor: Regarding “The Utility and Limitations of FRAX: A US Perspective”

**DOI:** 10.1007/s11914-012-0095-2

**Published:** 2012-02-01

**Authors:** Edward Czerwinski

**Affiliations:** Department of Bone and Joint Diseases, Medical College Jagiellonian University, ul. Kopernika 32, 31-501 Krakow, Poland

To the Editor:

In the publication: Stuart L. Silverman and Andrew D. Calderon. The Utility and Limitations of FRAX: A US Perspective (Curr Osteoporos Rep. 2010 December; 8(4): 192–197) on page 195, 2nd paragraph line 25–26, there is: “FRAX has recently been added to bone density software. It is currently on a calculator in Japan, a CD in Poland, and has now appeared on the US iPhone.”

**Fig. 1:**
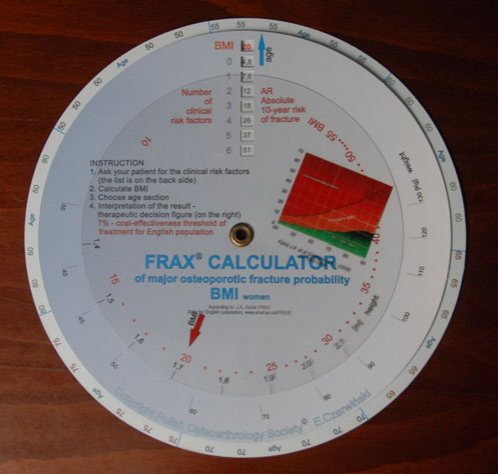
Hand-held FRAX calculator: general view on the side calculating fracture risk based on BMI

**Fig. 2:**
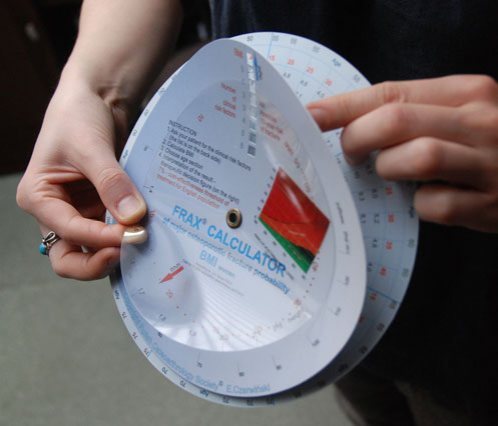
Hand-held FRAX calculator: the transparent disk with an *arrow* indicates BMI. This is calculated when weight is matched to height on the next disk

**Fig. 3:**
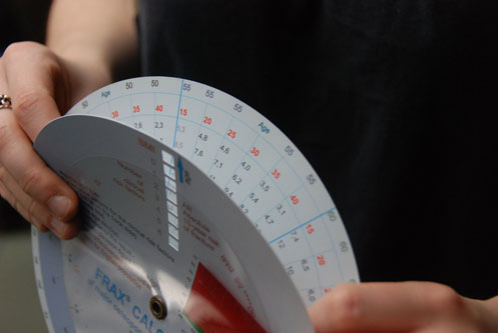
Hand-held FRAX calculator: on the inner disk there are fracture risk data according to FRAX (this is data for the English population). When BMI appears in the window (appropriate to the age) a value of fracture risk shows according to the number of clinical risk factors

**Fig. 4:**
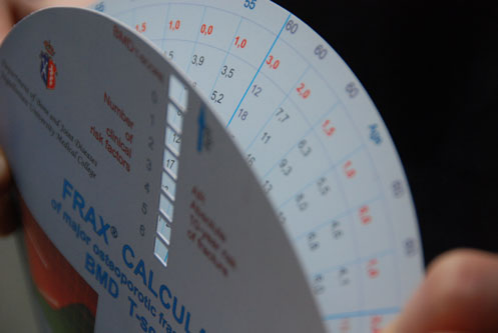
Hand-held FRAX calculator: On the outer disk there are numbers indicating risk factors. When the T-score appears in the window fracture risk may be read according to the number of risk factors

I wish to clarify that in Poland there is no FRAX on CD. Instead, in 2009 we developed a hand-held calculator which enables fracture risk calculation without using a computer. It consists of four independent paper disks with data and a window showing the result of calculations (Fig. 1). On one side BMI is calculated (known body weight and height), then shows 10-year risk of a fracture according to a number of risk factors (Fig. 2). The other side the calculator does the same when we know the T-score and the number of risk factors (Figs. 3 and 4).

This tool was devoted to medical staff and patients who have no access to FRAX online. I guess the author was misled by a slide frequently presented by Prof. John Kanis or Prof. Eugene McCloskey on which the tool looks like a CD but in fact it is not.

Below the text I’m enclosing a few photos presenting how it works and I am sending an original to you and the author by mail. This calculator is supposed to be used in the countries with poor or no access to Internet and it is based on a country’s epidemiological data. A study validating this tool against FRAX online is about to be published.

Best regards,

Prof. Edward Czerwinski

Head of the Department

